# Complete Mitochondrial Genome of Four Peristediidae Fish Species: Genome Characterization and Phylogenetic Analysis

**DOI:** 10.3390/genes15050557

**Published:** 2024-04-27

**Authors:** Xianhui Liao, Yijia Shih, Chenghao Jia, Tianxiang Gao

**Affiliations:** 1 Fisheries College, Zhejiang Ocean University, Zhoushan 316022, China; liaoxianhui@zjou.edu.cn; 2 Fisheries College, Jimei University, Xiamen 361021, China; eja0313@gmail.com; 3 School of Ecology and Environment, Hainan University, Haikou 570228, China; xicheng121@yeah.net

**Keywords:** Peristediidae, mitochondrial genome, phylogenetic analysis, cryptic species, kinship

## Abstract

The systematic revision of the family Peristediidae remains an unresolved issue due to their diverse and unique morphology. Despite the popularity of using mitochondrial genome research to comprehensively understand phylogenetic relationships in fish, genetic data for peristediid fish need to be included. Therefore, this study aims to investigate the mitochondrial genomic characteristics and intra-family phylogenetic relationships of Peristediidae by utilizing mitochondrial genome analysis. Therefore, this study aims to investigate the phylogenetic relationship of Peristediidae by utilizing mitochondrial genome analysis. The mitochondrial genome of four species of Peristediidae (*Peristedion liorhynchus*, *Satyrichthys welchi*, *Satyrichthys rieffeli*, and *Scalicus amiscus*) collected in the East China Sea was studied. The mitochondrial gene sequence lengths of four fish species were 16,533 bp, 16,526 bp, 16,527 bp, and 16,526 bp, respectively. They had the same mitochondrial structure and were all composed of 37 genes and one control region. Most PCGs used ATG as the start codon, and a few used GTG as the start codon. An incomplete stop codon (TA/T) occurred. The AT-skew and GC-skew values of 13 PCGs from four species were negative, and the GC-skew amplitude was greater than that of AT-skew. All cases of D-arm were found in *tRNA-Ser* (GCT). The Ka/Ks ratio analysis indicated that 13 PCGs were suffering purifying selection. Based on 12 PCGs (excluding ND6) sequences, a phylogenetic tree was constructed using Bayesian inference (BI) and maximum likelihood (ML) methods, providing a further supplement to the scientific classification of Peristediidae fish. According to the results of divergence time, the four species of fish had apparent divergence in the Early Cenozoic, which indicates that the geological events at that time caused the climax of species divergence and evolution.

## 1. Introduction

Family Peristediidae belongs to Scorpaeniformes and includes six valid genera: *Gargariscus* Smith (1917) (with a single nominal species); *Heminodus* Smith (1917) (with a single species); *Paraheminodus* Kamohara (1957) (with four valid species); *Peristedion* Lacepède (1801) (with 24 valid species; *Satyrichthys* Kaup (1873) (with 7 valid species); and *Scalicus* Jordan (1923) (with 8 valid species). Peristediidae are commonly known as armored searobins or armored gurnards. The peristediid fish are small to medium-sized benthic species, which have a wide roosting depth [1]. Peristedidea are covered with hard bone plates throughout the body, and each bone plate has spines on the body. They have various rostral projections, which is an important morphological feature that distinguishes them. Their rostral projection has four sensory pores, which can help them detect submerged prey in the sand, and the peristediid fish have two detached pectoral fins between the pectoral fin and pelvic fin. The detached pectoral fins can also help them find submerged prey in the sand [2]. In studies of peristediid systematics, the focus has been on morphological identification, and distinguishing characteristics have not always been clear within certain species [3]. Due to the strict fishing conditions and wide distribution range of Peristedidea, the incomplete species identification of Peristedidea and the inconsistent morphological results of some of them are also directions that need to be further studied. Therefore, revised systematics studies have been lacking in recent times.

The utilization of mitochondrial genomes (mtDNA) is a widely accepted method for comprehensively elucidating phylogenetic relationships within the fish group due to their characteristics such as small size, simple genomic structure, fast rate of evolution, and maternal inheritance with a low level of recombination [4]. The genome of mtDNA within general fish is similar to vertebrates within a circular molecule of 15~18 kb, which includes 13 protein-coding genes (PCGs), 2 ribosomal RNA (rRNA) genes, 22 transfer RNA (tRNA) genes, and a control region (CR) [5,6]. The control region includes a D-loop area and a light chain replication start area (OL). Whole-mtDNA genomes have been proven to be a valuable tool for understanding phylogenetic and molecular evolutionary studies across various taxonomic levels on a large scale [7,8,9]. In previous studies, the focus of fish species identification and systematics construction was primarily on individual genes within mtDNA, such as the ribosomal RNA (12S and 16S) and cytochrome c oxidase I (*COI*) [6,10,11], with only a few studies provided based on the mitochondrial genome [12]. Therefore, the enrichment of fish mitochondrial gene studies is of utmost importance. However, its application to members of the Peristeididae family is limited by the availability of data in existing databases. To date, it is unclear if these species’ mtDNA or genes were already annotated, and only the *S. amiscus* mitochondrial genome has been uploaded to the MitoFish GenBank in Peristediidae [13].

At present, there are few studies on the mitochondrial genome of Peristediidae, and there is no molecular phylogenetic relationship between Peristediidae in order to further explore the mitochondrial genome characteristics and phylogenetic relationships of Peristediidae. The complete mitochondrial genomes of four Peristediidae species (*P. liorhynchus*, *S. welchi*, *S. rieffeli*, and *S. amiscus*) were sequenced in this study. Subsequently, a comprehensive description and comparative analysis of the characteristics of the mtDNA genome were performed. Furthermore, an examination of the relative synonymous codon usage (RSCU) and AT skew value of PCGs was conducted to gain insights into the functional implications of associated genes. The phylogenetic relationships and divided time of peristediid fishes were ultimately calculated. The phylogenetic relationship conclusions of this study can be used to verify the accuracy of morphological classification results, and the mitochondrial genome results provide a molecular basis for subsequent further studies.

## 2. Materials and Methods

### 2.1. Sample Collection and Species Identification

**Figure 1:**
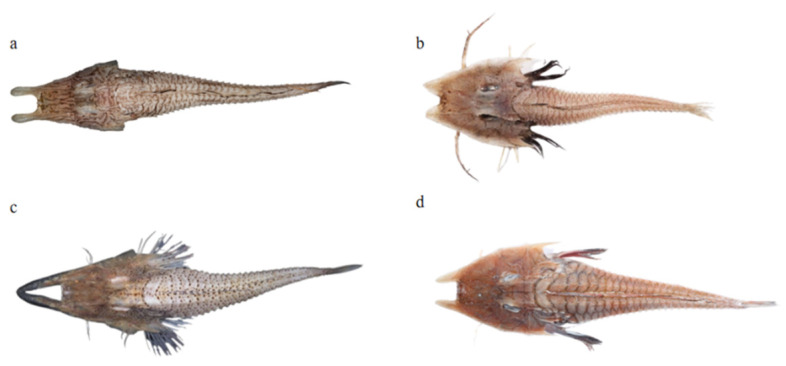
The samples of four Peristediidae fish species: (**a**) *P. liorhynchus*, (**b**) *S. amiscus*, (**c**) *S. rieffeli*, and (**d**) *S. welchi*.

More than 100 samples were collected in the East China Sea (127°–129° E, 28°–30° N) in December 2021. According to Kawai’s research, frozen samples were observed for their physical appearance and identified [2]. Four species (one sample per species) have been identified and confirmed through the process of identification (Figure 1). A small portion of the dorsal muscle was collected from each individual sample for DNA extraction. They were saved in liquid nitrogen and sent to the Wuhan Wanmo Technology Company for genome survey sequencing. All of the organisms (including experimental samples) were deposited in the Fisheries Ecology and Biodiversity Laboratory of Zhejiang Ocean University. Laboratory storage includes both specimen and DNA samples.

The DNA of the samples was tested for quality using 1% agarose electrophoresis for degradation and impurities, followed by the NanoPhotometer^®^ spectrophotometer (IMPLEN, CA, USA) for sample purity, and the Qubit^®^ 3.0 Flurometer (Life Technologies, Carlsbad, CA, USA) for DNA concentration. Qualified samples were processed into the library preparation process, including DNA fragmentation, end repair and addition of dA tails, adapter ligation, PCR amplification, PCR product detection, denaturation, and cyclization product testing. The constructed libraries were sequenced by PE using Illumina Nova. Finally, sequencing was performed, including DNB fabrication, DNB loading, and MGI platform sequencing. All data were sequenced by OneMore Technology (Wuhan, China).

### 2.2. Sequence Assembly, Annotation, and Analysis

Mitochondrial genome sequences were extracted from raw genome sequencing data using NOVOPlasty 2.6.3 software [14]. The parameters were set by default. The reference sequence was the single gene sequence corresponding to each species in GenBank. The obtained data was annotated and visualized using MitoFish to obtain a complete mitochondrial genome [15]. Using MEGA 11 to calculate genomic base composition, amino acid usage frequency, and relative usage frequency of synonymous codons [16].

### 2.3. Phylogenetic Analyses and Divergence Times Analyses

The mitochondrial genomes of 10 Scorpaeniformes species were downloaded from GenBank (MT855984.1, MN122888.1, KY379222.1, KY012348.1, MZ442599.1, LC493914.1, MT801083.1, MK784116.1, MN104592.1). Phylogenetic analysis based on 12 protein-coding genes (excluding *ND6*). The *ND6* gene was not used in this analysis because of a significant anti-G shift. *Dactylopterus volitans* (GenBank accession number: LC512458.1) was used as an outgroup to determine the classification relationship of Peristediidae. The Bayesian inference (BI) and maximum likelihood (ML) methods were used and the optimal model for nucleotide sequences was estimated using jModelTest v2.1.10 [17]. The model GTR + G + I captured the minimum values of the Bayesian Information Criterion (BIC, estimation 67) and was considered to be the best model for phylogenetic tree construction [18]. The ML tree was constructed using MEGA 11 software with 1000 replicates of bootstrapping and the BI analysis was inferred by the software of MrBayes v3.2.7a [19]. The divergence time was estimated using MEGA 11.0 with the RelTime-ML method and GTR + G + I modeling [20]. The calibration of divergence times was obtained from the online Time Tree database [21].

All the above analysis methods used default parameters for calculation, except for the specific parameters mentioned.

## 3. Results

### 3.1. Genome Structure and Nucleotide Composition

**Figure 2:**
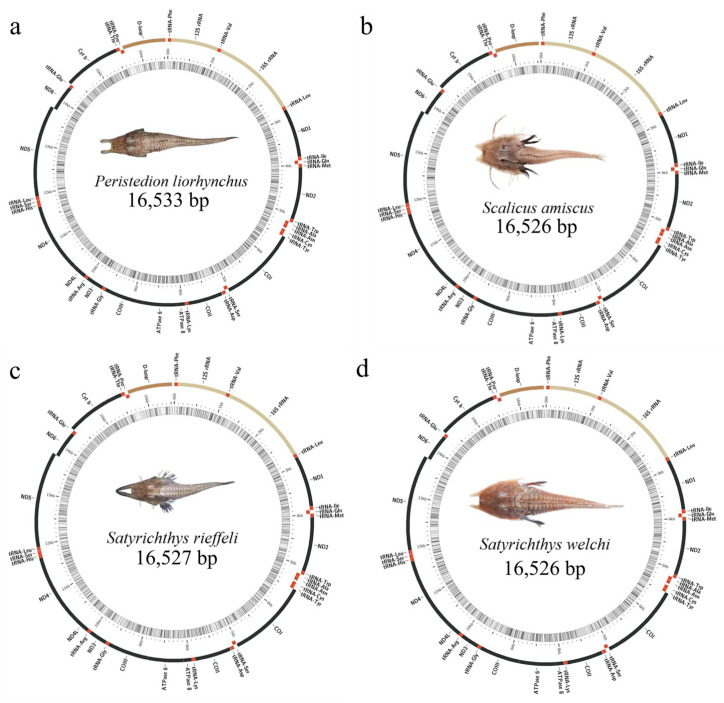
The circular map of the mitogenome of four Peristediidae fishes. (**a**): *P. liorhynchus*, (**b**): *S. amiscus*, (**c**): *S. rieffeli*, and (**d**): *S. welchi*.

The mitochondrial sequences obtained in this study were as follows: *P. liorhynchus*, *S. welchi*, *S. rieffeli*, and *S. amiscus* had lengths of 16,533 bp, 16,526 bp, 16,527 bp, and 16,526 bp, respectively. All of the mitogenome was composed of 37 genes and one control region, including 13 PCGs (*ATP6*, *ATP8*, *Cytb*, *COXI-III*, *ND1-6*, and *ND4L*), two rRNA genes (*12S rRNA* and *16S rRNA*), 22 tRNA genes, and one D-loop region (Tables S1–S4 and Figure 2). ND6 and 8 tRNA genes (*Gln*, *Ala*, *Asn*, *Cys*, *Tyr*, *Ser*, *Glu*, and *Pro*) were encoded on the light strand (L-strand), and the others were encoded on the heavy strand (H-strand). The D-loop regions were encoded between the *tRNA-Pro* and *tRNA-Phe* genes. The gene structure and arrangement of these species exhibited no instances of gene rearrangement. *P. liorhynchus* found 6 overlapping regions and 10 intergenic spacer regions, totaling 24 and 65 bp, respectively. *S. welchi* found 6 overlapping regions and 10 intergenic spacer regions, totaling 24 and 67 bp, respectively. *S. rieffeli* found 4 overlapping regions and 11 intergenic spacer regions, totaling 8 and 99 bp, respectively. *S. amiscus* found 7 overlapping regions and 10 intergenic spacers regions, a total of 25 and 65 bp, respectively. The overall base composition was 26.7–27.4% for A, 28.6–30% for C, 16.7–17.2% for G, and 26–27.3% for T (Table S5). Four species had low levels of G and had a clear bias towards G. Additionally, the third codon position exhibited a high preference for A and C in terms of codon usage, as evidenced by the elevated content levels observed in both nucleotides A and C. The A + T content of the whole mitogenome ranged from 52.8% to 54.4%. All regions of the whole mitogenome exhibited a higher A + T content compared to the G + C content.

### 3.2. Protein-Coding Genes (PCGs)

The lengths of 13 PCGs in *P. liorhynchus*, *S. welchi*, *S. rieffeli*, and *S. amiscus* were 10,578 bp, 10,577 bp, 10,541 bp, and 10,577 bp, respectively. The H-strand of the mitochondrial genome encoded 12 PCGs in each species, while only the *ND6* gene was expressed on the L-strand. Among them, there were 12 PCGs of 3 species (*S. welchi*, *S. rieffeli*, and *S. amiscus*) initiated with ATG as the start codon, while *COI* initiated with GTG as the start codon like other bony fish. For the remaining species, 11 PCGs started with ATG as the start codon, while *COI* and *ATPase 6* genes initiated with GTG. Four complete termination codons (TAA or TAG) were identified in each species, while incomplete termination codons (TA or T) were also detected. TA was detected in *ND2*, *ATPase 6* and *COIII* in 5 species (*S. welchi*, *S. rieffeli*, *S. amiscus*, *Paraheminodus laticephalus*, and *Paraheminodus murrayi*). In addition to these genes, TA was also detected in *ND4* in one species (*P. liorhynchus*). T was detected in *COII*, *ND3*, *ND4*, and *Cyt b* in three species, while the remaining ones were detected in *COII*, *ND3*, and *Cyt b*.

**Figure 3:**
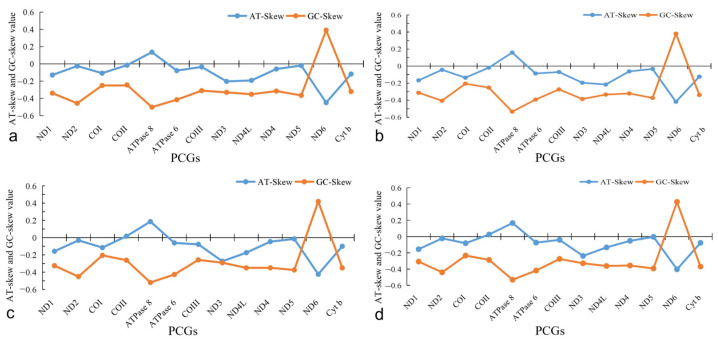
AT-skew and GC-skew values for four species. (**a**): *P. liorhynchus*, (**b**): *S. amiscus*, (**c**): *S. rieffeli*, and (**d**): *S. welchi*.

Most of the AT-skew and GC-skew values for the four species were negative (Figure 3). The abundance of base T and C exceeded that of A and G, while the scarcity of base T and C was more pronounced compared to A and G. Overall, the magnitude of GC-skew was consistently greater than AT-skew. The maximum difference in the *ND6* gene was observed in all four species.

### 3.3. RNA Genes

In the mitogenomes of the four species, a total of 22 tRNA genes were identified. Among them, *tRNA-Leu* (TAA, TAG) and *tRAN-Ser* (GCT, TGA) possessed two types of anticodons, while others had only one type. Eight tRNA genes were located on the L-stand and fourteen on the H-stand. The structure of *tRNA-Ser* (GCT) was characterized by a simple loop lacking a dihydrouridine arm (D-arm), whereas all other tRNAs exhibited canonical cloverleaf secondary structure (Figure S1). The base composition for these genes was as follows: 26.3–26.8% for T, 21.4–22.0% for C, 27.6–28.0% for A, and 23.8–24.2% for G.

The H-strand exhibited the presence of *12S rRNA* and *16S rRNA*. Especially, the *12S rRNA* gene was positioned between *tRNA-Phe* and *tRNA-Val*, while the *16S rRNA* gene resided between *tRNA-Val* and *tRNA-Leu*. The length range of the *12S rRNA* gene was determined to be between 945 bp and 946 bp, while that of the *16S rRNA* gene was found to be between 1695 bp and 1699 bp.

### 3.4. Usage of Mitogenome Codon

**Figure 4:**
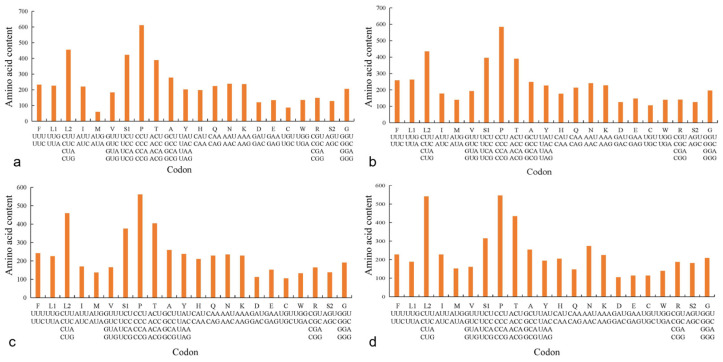
Codon frequency of the mitochondrial genome for four species: (**a**) *P. liorhynchus*, (**b**) *S. amiscus*, (**c**) *S. rieffeli*, and (**d**) *S. welchi*. Note: F(*Phe*), L1(*Leu*), L2(*Leu*), I(*Ile*), M(*Met*), V(*Val*), S1(*Ser*), P(*Pro*), T(*Thr*), A(*Ala*), Y(*Tyr*), H(*His*), Q(*Gln*), N(*Asn*), K(*Lys*), D(*Asp*), E(*Glu*), C(*Cys*), W(*Trp*), R(*Arg*), S2(*Ser*), G(*Gly*).

**Figure 5:**
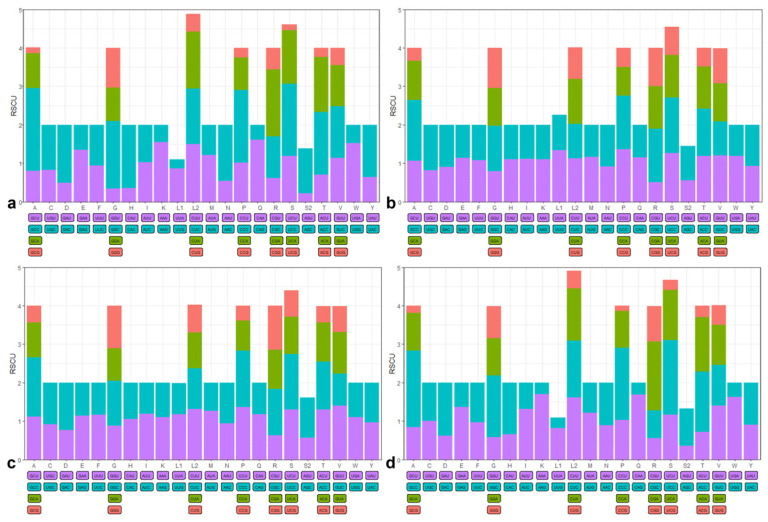
Relative synonymous codon usage (RSCU) of the mitochondrial genome for four species: (**a**) *P. liorhynchus*, (**b**) *S. amiscus*, (**c**) *S. rieffeli*, and (**d**) *S. welchi*. Note: F(*Phe*), L1(*Leu*), L2(*Leu*), I(*Ile*), M(*Met*), V(*Val*), S1(*Ser*), P(*Pro*), T(*Thr*), A(*Ala*), Y(*Tyr*), H(*His*), Q(*Gln*), N(*Asn*), K(*Lys*), D(*Asp*), E(*Glu*), C(*Cys*), W(*Trp*), R(*Arg*), S2(*Ser*), G(*Gly*).

The amino acid *tRNA-Leu* exhibited the highest codon usage value, being utilized by six distinct codons. Conversely, the frequencies of *tRNA-Asp* and *tRNA-Cys* were relatively low, with each employed by only two different codons (Figure 4). The RSCU value of all amino acids in the four species was not uniformly equal to 1, indicating varying degrees of bias in the utilization of each amino acid (Figure 5).

### 3.5. Ka/Ks

**Figure 6:**
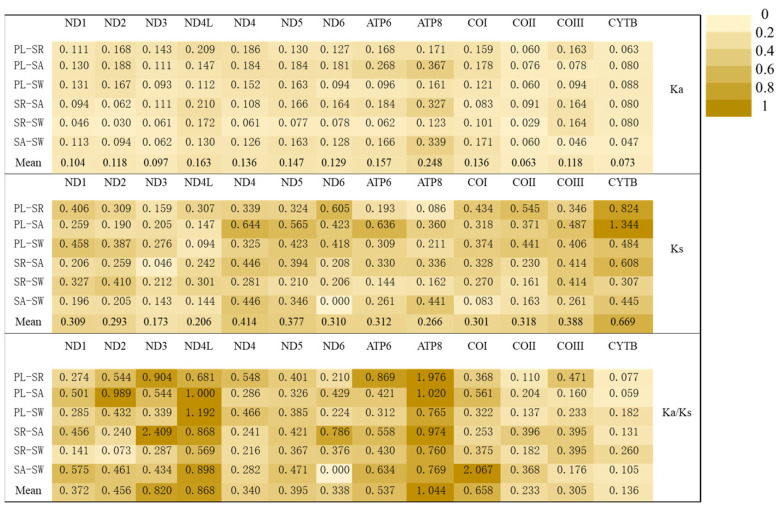
The Ka/Ks values for each protein-coding gene (PCG) in the pairwise mitochondrial genomes of four peristediid fishes. The abbreviations used are as follows: (PL) *P. liorhynchus*, (SW) *S. welchi*, (SR) *S. rieffeli*, and (SA) *S. amiscus*.

In this study, the value of Ka/Ks greatly deviated (Figure 6). Positive selection mainly occurred between different species in the *ND4L* and *ATP8* genes. Among them, the values of the *ND3* gene were significantly greater than 1 in *S. rieffeli* and *S. amiscus*, and the values of the COI gene were significantly greater than 1 in *S. amiscus* and *S. welchi*. The *ND1*, *ND4*, *ND5*, *COII*, *COIII*, and *Cyt-b* genes were only selected for purification and at small values.

### 3.6. Phylogenetic and Divergence Times

**Figure 7:**
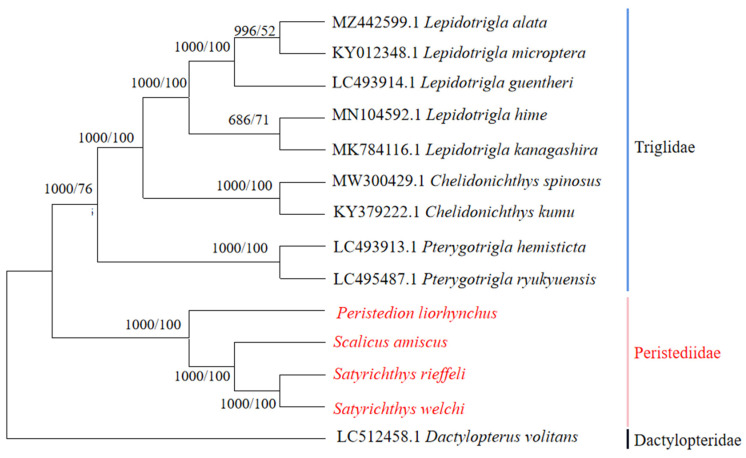
Phylogenetic tree based on the Bayesian (BI) and maximum likelihood (ML) methods (based on 12 PCGs). *D. volitans* was used as the outgroup.

Fourteen species, belonging to three families (Peristediidae, Triglidae, and Dactylopteridae), were included in the analysis of topology and divergence times. *D. volitans* was selected as the outgroup (Figure 7). The cladograms generated by both BI and ML methods exhibited consistent results, providing clear evidence for the distinct separation of these two families. Both families, Peristediidae and Triglidae, were sister lines. Within the family Peristediidae, the genus *Peristedion* was distinguished as the first sub-clade, followed by the separation of the genus *Satyrichthys* from other genera.

**Figure 8:**
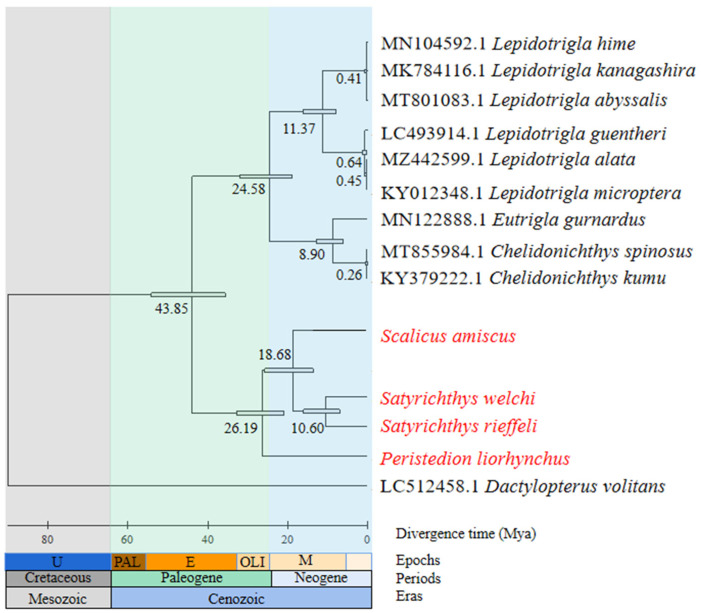
Based on the maximum likelihood (ML) topology, the divergent time trees of 14 fish species were constructed, and the tandem sequences of 12 PCGs were used. The number near the node represents the estimated divergence time (Mya).

The divergence time calculations showed that the earliest divergence between Triglidae and Peristediidae occurred 43.85 million years ago (Mya) (Figure 8). In the family Peristediidae, *P. liorhynchus* was the earliest to differentiate from the other Peristediidae (26.19 Mya). According to the estimated divergence time, these two families, Peristediidae and Triglidae, underwent speciation during the Cenozoic era. Both families belong to the suborder Triglioidei, which first appeared in the Late Cretaceous–Early Paleogene period.

## 4. Discussion

The mitochondrial genomes of four Peristediidae species were all composed of 37 genes and one control region. The gene structure and arrangement of these species were identical to those of other vertebrate mitogenomes, and none of them exhibit genetic rearrangements. The gene rearrangement is always detected in fungi and insects [22,23,24,25]. The conservation of the mitochondrial genome is widely acknowledged, making gene rearrangement a highly studied feature in organism mtDNAs for organismal systematics [26,27]. The rearrangements of mitochondrial genes can generally be associated with genomic variation, aspects of physiology, molecular mechanism, life history, or genomic evolutionary processes [3,28,29]. The evidence suggests that the mtDNAs of Peristediidae fish may exhibit a higher degree of conservation compared to other members within the class Actinopterygii. Therefore, an understanding of the arrangement and combination of these nucleotides among species, as well as the statistical results, is of great significance for further exploration of the splitting of species. *ND6* and 8 tRNA genes (*Gln*, *Ala*, *Asn*, *Cys*, *Tyr*, *Ser*, *Glu*, and *Pro*) were encoded on the light strand (L-strand), and the others were encoded on the heavy strand (H-strand). The D-loop region was encoded between the *tRNA-Pro* and *tRNA-Phe* genes. The base content of all six species appeared to have a significant anti g bias. It was related to the hydrophobic character of proteins [30].

For the initiation codon in the *COI* gene, all four species utilized GTG, which is consistent with findings from previous studies on other bony fish [31]. Besides, *S. amiscus* used GTG as the initiation codon in the *ATP 6* gene. Six complete termination codons (TAA or TAG) for each species were detected. Moreover, incomplete termination codons (TA or T) were discovered. The presence of incomplete stop codons is common in fish mitochondrial genomes. In fish genetics, this can be achieved by adding poly A tails during RNA processing [32]. The phenomenon of incomplete termination codons was discovered in this study, which is significant for further investigation.

The AT-skew and GC-skew have been regarded as potential indicators for quantifying strand asymmetry and nucleotide composition patterns. The majority of genes in this study exhibited a higher magnitude of GC-skew compared to AT-skew, aligning with conventional preferences that favor a more pronounced GC-skew [33]. However, the *ND6* gene was observed to have the highest difference between the GC-skew values and AT-skew values, which was consistent with the significant differences in the AT-skew and GC-skew values of *Cheilinus undulatus* in the DN6 gene [34]. The *ND6* gene had a larger fluctuation in AT/GC-skew value, suggesting that the selection and mutational pressure on it might be significantly different from other genes [33].

The secondary structure of all 21 tRNAs in the four species exhibited a canonical cloverleaf pattern, and the *tRNA-Ser* (GCT) displayed a simplified loop without the dihydrouridine arm (D-arm). This characteristic is commonly observed in the mitogenomes of many bony fish [35]. For bony fish, the missing D-arm could be transformed into the recognition potential to be recognized [33]. The *12s rRNA* and *16s rRNA* were divided by *tRNA-Val* and located between *tRNA-Phe* and *tRNA-Leu* on the H-strand. Miya exhibited the same structure in that study of the mitochondrial genome of round and pointed-head grenadier fishes [36]. The same structure occurs in *Sebastiscus marmoratus* [37].

The RSCU method was also employed for the evaluation of codon usage in mitochondrial genes. Distinct RSCU values indicated diverse scenarios of codon usage [38]. The RSCU values of four species were found to be unequal to 1, indicating that the utilization of each amino acid exhibited varying degrees of bias. Among the codon usage of the four species, NNT and NNC were more used, and Leu > Pro > Ser > Thr. The codon use bias is related to the strength of gene expression, and the codons used by the genes with high efficiency expression have significantly different gene use frequencies compared with the genes with low expression. The genes with efficient expression have more bias in the use of codons than the non-efficient genes, and they usually use a set of preferential synonymous codons [39]. The result suggested that the gene function might be similar within the family Peristediidae. The main factors influencing codon bias include mutation pressure, genetic drift, and natural selection [40].

The value of non-synonymous substitution (Ka)/synonymous substitution (Ks) is a common indicator for assessing the relationship between species selection pressure and evolution in molecular studies. Ka/Ks < 1, Ka/Ks = 1, and Ka/Ks > 1 represent purified selection, neutral mutation, and positive selection, respectively. The ratio of Ka/Ks is generally considered an indicator of selective pressure and evolutionary relationships at the molecular level among homogenous or heterogeneous species [41]. In this study, the genes that were obviously in positive selection (*ND4L* and *ATP8*) also showed Ka/Ks values greater than 1 (*COI* and *ND3*), and the Ka/Ks values of the remaining genes were less than 1, but much larger than those of fish genes that were normally in purification selection [42]. The higher positive selection for the *ND4L* and *ATP8* genes may be due to the fact that their gene sequences are shorter, which makes them more susceptible to genetic mutations and drift. The Ka/Ks value is greater than 1, and one of the reasons for this result is that gene mutations and drift caused by the environment lead to genome evolution [43]. Peristediidae fish live in a deep water depth of about 40–1000 m at the bottom of the demersal fish. Demersal fish live in conditions such as lack of oxygen, lack of food, lack of sunlight, and extreme cold. Such an environment is the cause of Ka/Ks > 1 [44]. The root cause of positive selection is often related to environmental adaptation and the development of new functions, and most non-synonymous mutations are unfavorable [43]. Compared to other genes, the *ND4L* and *ATP8* genes had a higher average value in each pair comparison. Due to DNA repair mechanisms, low mutation rates tend to occur in highly expressed genes, and *COII* and *Cytb* have lower Ka/Ks levels and lower mutation rates compared with other genes, suggesting that they may have higher expression levels [45]. Fish of the same genus showed lower Ka/Ks values in different genes, indicating that fish of the same genus showed similar living habits and had a closer phylogenetic relationship [42]. Peristediidae fishes are demersal fishes, and their living environment extends from the bottom of the front sea to the bottom of the deep sea. Their living environment is the same, and their morphological characteristics are generally the same [2].

The topology tree results obtained by the BI and ML methods consistently indicate the two families, Peristediidae and Triglidae. The result was consistent with the traditional systematics based on morphological characteristics [2]. Furthermore, the analysis reveals that the genus *Peristedion* exhibits the farthest genetic divergence within the family Peristediidae.

In the family Peristediidae, the divergence time between the genus *Peristedion* and other species within Peristediidae was the earliest (26.19 Mya). Conversely, the genera *Scalicus* and *Paraheminodus* exhibited a more recent divergence time of 4.58 Mya. Notably, there existed a significant gap of 7.51 million years between the genera *Peristedion* and *Satyrichthys*. The results of this study suggest that reproductive isolation and evolutionary processes in different genera occurred over a long period of time. These findings underscore the long-term nature of evolution, aligning with previous research [46]. Additionally, alterations in genetic structure can also result in reproductive isolation among organisms. The occurrence of geological events constitutes the primary factor leading to geographical isolation [47,48]. The divergence time of species in the family Peristediidae can be traced back to the late Cretaceous period (66–145 Mya). A multitude of geological transformations have transpired during this time period. The formation of numerous mountain ranges, the emergence of flowering plants, and a substantial accumulation of shale deposits on the ocean floor have contributed to the manifestation of two marine anoxic events [49]. The occurrence of these events has resulted in changes to the fish habitat, leading to their divergence in this region. During the Cenozoic–Mesozoic period, which occurred approximately 240–65 Mya, there was a significant reduction in transgressive range on the continent and the emergence of marine sediments in marginal areas of China [50]. During this period, there were frequent occurrences of geological movements. These geological changes have the potential to generate an increased abundance of food sources for fish and can also result in geological isolation, thereby altering genetic trajectories. The findings from this study reveal that a majority of fish species underwent differentiation during the Early Cenozoic, further substantiating the notion that geological events exerted a discernible influence on species divergence.

## 5. Conclusions

In this study, mitochondrial genome sequence analysis was used to explore four Peristiidae species. The complete mitochondrial genome sequences and mitochondrial gene structures of four Peristiidae species were determined. The structure remains consistent, encompassing a total of 37 genes and one control region. The cladogram was constructed using two methods to determine the classification of the family Peristiidae. The *P. liorhynchus* was preferentially separated, the *S. rieffeli* and the *S. welchi* were gathered into one branch, and the *S. amiscus* and *Satyrichthys* were sister branches. The results of the classification within the family were consistent with those obtained from morphological studies. The divergence time tree and timeline were constructed based on the ML tree. The four peristiid species underwent differentiation during the Early Paleogene period, and their evolution trajectory may be linked to geological events that could have altered their habitat conditions. The results of this study provide a basic theoretical and molecular basis for the taxonomic study of Peristiidae. The complete mitogenome of these four peristiid fishes will provide crucial insights for enhancing the taxonomic system and phylogenetics.

## Data Availability

The datasets generated during this study have been uploaded to NCBI (Nos. PP708603; PP708604; PP708605; PP708606).

## Supplementary Materials

The following supporting information can be downloaded at: https://www.mdpi.com/article/10.3390/genes15050557/s1, Table S1. Summary of gene/element feature of *P. liorhynchus*; Table S2: Summary of gene/element feature of *S. welchi*; Table S3: Summary of gene/element feature of *S. rieffeli*; Table S4: Summary of gene/element feature of *S. amiscus*; Table S5: The base composition of four *Peristediidae fish*; Figure S1. Inferred secondary structures of *tRNA-Ser* (GCT) gene in four *Peristediidae mitogenomes*.
