# Supplementary material for: Complete Mitochondrial Genome of Four Peristediidae Fish Species: Genome Characterization and Phylogenetic Analysis
Source: Genes (Basel). 2024 Apr 27;15(5):557. doi: 10.3390/genes15050557 (PMC11121196; doi:10.3390/genes15050557)
Supplement: Supplementary file 1 [file genes-15-00557-s001.zip › Supplementary Materials.pdf]

Table S1. Summary of gene/element feature of *P. liorhynchus*.

Gene	Position-start	Position-end	Length (bp)	Amino acid	Start codon	Stop codon	Intergenic region (bp)	Strand
tRNA-Phe	1	68	68				0	H
12S rRNA	69	1014	946				0	H
tRNA-Val	1015	1086	72				0	H
16S rRNA	1087	2781	1695				0	H
tRNA-Leu	2782	2855	74				0	H
ND1	2856	3830	975	324	ATG	TAG	0	H
tRNA-Ile	3835	3904	70				4	H
tRNA-Gln	3904	3974	71				-1	L
tRNA-Met	3974	4042	69				-1	H
ND2	4043	5088	1046	348	ATG	TA	0	H
tRNA-Trp	5089	5159	71				0	H
tRNA-Ala	5161	5229	69				1	L
tRNA-Asn	5231	5303	73				1	L
tRNA-Cys	5341	5406	66				37	L
tRNA-Tyr	5407	5476	70				0	L
COI	5478	7028	1551	516	GTG	TAA	1	H
tRNA-Ser	7029	7099	71				0	L
tRNA-Asp	7103	7175	73				3	H
COII	7183	7873	691	230	ATG	T	7	H
tRNA-Lys	7874	7947	74				0	H
ATPase 8	7949	8116	168	55	ATG	TAA	1	H
ATPase 6	8107	8789	683	227	ATG	TA	-10	H
COIII	8790	9574	785	261	ATG	TA	0	H
tRNA-Gly	9575	9646	72				0	H
ND3	9647	9995	349	116	ATG	T	0	H
tRNA-Arg	9996	10064	69				0	H
ND4L	10065	10361	297	98	ATG	TAA	0	H
ND4	10355	11736	1382	460	ATG	TA	-7	H
tRNA-His	11737	11805	69				0	H
tRNA-Ser	11806	11873	68				0	H
tRNA-Leu	11879	11951	73				5	H
ND5	11952	13790	1839	611	ATG	TAA	0	H
ND6	13787	14308	522	173	ATG	TAG	-4	L
tRNA-Glu	14309	14377	69				0	L
Cyt b	14383	15523	1141	380	ATG	T	5	H
tRNA-Thr	15524	15595	72				0	H
tRNA-Pro	15595	15664	70				-1	L
D-loop	15665	16533	869				0	H

Table S2. Summary of gene/element feature of *S. welchi.*

Gene	Position-start	Position-end	Length (bp)	Amino acid	Start codon	Stop codon	Intergenic region (bp)	Strand
tRNA-Phe	1	68	68				0	H
12S rRNA	69	1014	946				0	H
tRNA-Val	1015	1086	72				0	H
16S rRNA	1087	2784	1698				0	H
tRNA-Leu	2785	2858	74				0	H
ND1	2859	3833	975	324	ATG	TAA	0	H
tRNA-Ile	3838	3907	70				4	H
tRNA-Gln	3907	3977	71				-1	L
tRNA-Met	3977	4045	69				-1	H
ND2	4046	5091	1046	348	ATG	TA	0	H
tRNA-Trp	5092	5162	71				0	H
tRNA-Ala	5164	5232	69				1	L
tRNA-Asn	5234	5306	73				1	L
tRNA-Cys	5346	5411	66				39	L
tRNA-Tyr	5412	5481	70				0	L
COI	5483	7033	1551	516	GTG	TAA	1	H
tRNA-Ser	7034	7104	71				0	L
tRNA-Asp	7108	7180	73				3	H
COII	7188	7878	691	230	ATG	T	7	H
tRNA-Lys	7879	7952	74				0	H
ATPase 8	7954	8121	168	55	ATG	TAA	1	H
ATPase 6	8112	8794	683	227	ATG	TA	-10	H
COIII	8795	9579	785	261	ATG	TA	0	H
tRNA-Gly	9580	9651	72				0	H
ND3	9652	10000	349	116	ATG	T	0	H
tRNA-Arg	10001	10069	69				0	H
ND4L	10070	10366	297	98	ATG	TAA	0	H
ND4	10360	11740	1381	460	ATG	T	-7	H
tRNA-His	11741	11809	69				0	H
tRNA-Ser	11810	11877	68				0	H
tRNA-Leu	11883	11955	73				5	H
ND5	11956	13794	1839	612	ATG	TAA	0	H
ND6	13791	14312	522	173	ATG	TAG	-4	L
tRNA-Glu	14313	14381	69				0	L
Cyt b	14387	15527	1141	380	ATG	T	5	H
tRNA-Thr	15528	15599	72				0	H
tRNA-Pro	15599	15668	70				-1	L
D-loop	15669	16526	858				0	H

Table S3. Summary of gene/element feature of *S. rieffeli.*

Gene	Position-start	Position-end	Length (bp)	Amino acid	Start codon	Stop codon	Intergenic region (bp)	Strand
tRNA-Phe	1	68	68				0	H
12S rRNA	69	1014	946				0	H
tRNA-Val	1015	1086	72				0	H
16S rRNA	1087	2785	1699				0	H
tRNA-Leu	2786	2859	74				0	H
ND1	2860	3834	975	324	ATG	TAG	0	H
tRNA-Ile	3839	3908	70				4	H
tRNA-Gln	3908	3978	71				-1	L
tRNA-Met	3978	4046	69				-1	H
ND2	4047	5092	1046	348	ATG	TA	0	H
tRNA-Trp	5093	5163	71				0	H
tRNA-Ala	5165	5233	69				1	L
tRNA-Asn	5235	5307	73				1	L
tRNA-Cys	5347	5412	66				39	L
tRNA-Tyr	5413	5482	70				0	L
COI	5484	7034	1551	516	GTG	TAA	1	H
tRNA-Ser	7035	7105	71				0	L
tRNA-Asp	7109	7181	73				3	H
COII	7189	7879	691	230	ATG	T	7	H
tRNA-Lys	7880	7953	74				0	H
ATPase 8	7955	8122	168	55	ATG	TAA	1	H
ATPase 6	8113	8795	683	227	ATG	TA	0	H
COIII	8796	9580	785	261	ATG	TA	0	H
tRNA-Gly	9581	9652	72				0	H
ND3	9653	10001	349	116	ATG	T	0	H
tRNA-Arg	10002	10070	69				0	H
ND4L	10071	10367	297	98	ATG	TAA	0	H
ND4	10361	11741	1381	460	ATG	T	-5	H
tRNA-His	11742	11810	69				0	H
tRNA-Ser	11811	11878	68				0	H
tRNA-Leu	11884	11956	73				5	H
ND5	11957	13759	1803	600	ATG	TAA	0	H
ND6	13792	14313	522	173	ATG	TAG	32	L
tRNA-Glu	14314	14382	69				0	L
Cyt b	14388	15528	1141	380	ATG	T	5	H
tRNA-Thr	15529	15600	72				0	H
tRNA-Pro	15600	15669	70				-1	L
D-loop	15670	16527	858				0	H

Table S4. Summary of gene/element feature of *S. amiscus*.

Gene	Position-start	Position-end	Length (bp)	Amino acid	Start codon	Stop codon	Intergenic region (bp)	Strand
tRNA-Phe	1	68	68				0	H
12S rRNA	69	1014	946				0	H
tRNA-Val	1015	1086	72				0	H
16S rRNA	1087	2784	1698				0	H
tRNA-Leu	2785	2858	74				0	H
ND1	2859	3833	975	324	ATG	TAG	0	H
tRNA-Ile	3838	3907	70				4	H
tRNA-Gln	3907	3977	71				-1	L
tRNA-Met	3977	4045	69				-1	H
ND2	4046	5091	1046	348	ATG	TA	0	H
tRNA-Trp	5092	5162	71				0	H
tRNA-Ala	5164	5232	69				1	L
tRNA-Asn	5234	5306	73				1	L
tRNA-Cys	5346	5412	67				39	L
tRNA-Tyr	5413	5482	70				0	L
COI	5484	7034	1551	516	GTG	TAA	1	H
tRNA-Ser	7035	7105	71				0	L
tRNA-Asp	7109	7181	73				3	H
COII	7189	7879	691	230	ATG	T	7	H
tRNA-Lys	7880	7953	74				0	H
ATPase 8	7955	8122	168	55	ATG	TAA	1	H
ATPase 6	8113	8795	683	227	GTG	TA	-10	H
COIII	8796	9580	785	261	ATG	TA	0	H
tRNA-Gly	9581	9652	72				0	H
ND3	9653	10001	349	116	ATG	T	0	H
tRNA-Arg	10001	10070	70				-1	H
ND4L	10071	10367	297	98	ATG	TAA	0	H
ND4	10361	11741	1381	460	ATG	T	-7	H
tRNA-His	11742	11810	69				0	H
tRNA-Ser	11811	11878	68				0	H
tRNA-Leu	11885	11957	73				6	H
ND5	11958	13796	1839	612	ATG	TAG	0	H
ND6	13793	14314	522	173	ATG	TAG	-4	L
tRNA-Glu	14315	14383	69				0	L
Cyt b	14389	15529	1141	380	ATG	T	5	H
tRNA-Thr	15530	15601	72				0	H
tRNA-Pro	15601	15670	70				-1	L
D-loop	15671	16526	856				0	H

Table S5. The base composition of 4 Peristediidae fishes.

	Pe.P. liorhynchus				*Sa. welchi*				*Sa. rieffeli*				*Sc. amiscus*			
	T	C	A	G	T	C	A	G	T	C	A	G	T	C	A	G
ND1	29.4	32	22.7	15.8	30.8	30.6	22.5	16.2	30.8	31	22.4	15.8	31.2	30.5	22.3	16.0
ND2	25.4	36.7	24.2	13.7	27.5	33.2	26.3	12.9	28.0	33.1	26.3	12.5	28.0	32.6	25.7	13.8
COI	28.4	30.5	22.9	18.3	29.1	28.5	24.7	17.7	30.5	27.3	24.2	18.0	31.1	27.3	23.7	18.0
COII	27.8	28.1	27	17.1	27.0	28.7	28.4	15.9	27.4	28.0	28.3	16.4	28.8	27.1	27.8	16.2
ATPase 8	21.2	38.2	27.9	12.7	21.2	37.6	29.7	11.5	21.2	36.4	30.9	11.5	22.4	35.8	30.9	10.9
ATPase 6	27.9	34.1	23.9	14.1	28.8	32.9	24.8	13.5	28.6	32.9	25.3	13.2	29.7	31.6	25.0	13.8
COIII	26.7	31.7	24.9	16.7	26.9	30.8	24.8	17.5	28.4	29.8	24.3	17.6	28.1	30.1	24.5	17.2
ND3	28.2	35.3	18.7	17.8	32.2	31.9	19.8	16.1	32.8	31.3	18.7	17.2	30.7	33.6	20.7	14.9
ND4L	29.6	34.0	20.1	16.3	28.9	33.3	22.1	15.6	28.6	34.7	20.1	16.7	31.6	32.0	20.4	16.0
ND4	27.9	31.1	24.8	16.2	28.1	31.6	25.3	15.0	28.3	30.9	25.8	14.9	28.4	30.7	25.1	15.8
ND5	27.2	31.8	26.2	14.8	27.2	31.9	27.0	13.9	27.8	31.0	27.0	14.1	28.6	30.6	26.8	14.0
ND6	36.3	15.2	13.8	34.7	37.6	13.3	16.0	33.1	37.6	13.7	15.2	33.5	36.0	15.2	14.8	33.9
Cyt b	28.6	32.2	22.6	16.6	27.8	33.2	23.8	15.3	29.1	31.8	23.8	15.3	29.6	31.7	23.1	15.7
PCGs	28.0	31.6	23.1	17.3	28.7	30.6	24.2	16.5	29.2	30.0	24.6	16.2	29.6	29.9	23.9	16.6
rRNA	21.1	25.6	32.6	20.7	21.8	25.6	31.7	21.0	21.9	25.3	31.9	20.9	22.2	24.8	31.7	21.3
tRNA	26.3	22.0	27.7	24.0	26.5	21.8	27.9	23.8	26.7	21.4	27.6	24.2	26.8	21.4	28.0	23.8
CR	30.1	21.2	31.8	16.9	29.6	21.7	31.7	17.0	30.0	21.4	32.3	16.3	29.7	20.9	32.1	17.3
Mitogenome	26.0	30.0	26.8	17.2	26.5	29.4	27.4	16.7	26.9	28.9	27.4	16.8	27.3	28.6	27.1	17.0

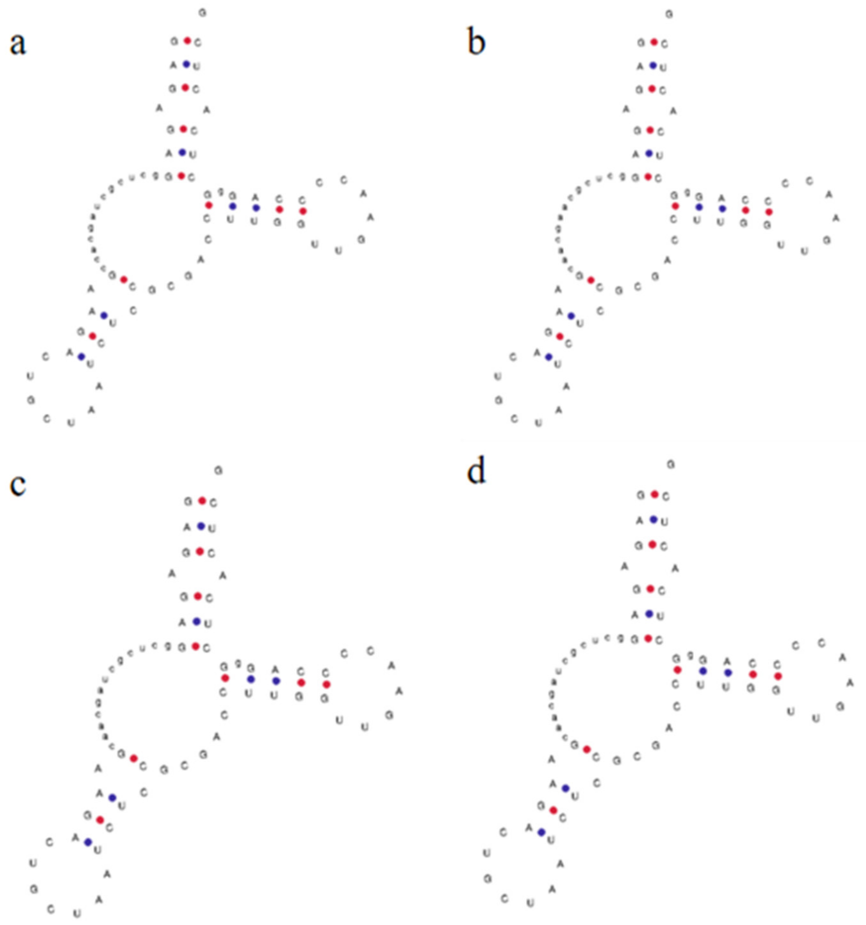

Figure S1. Inferred secondary structures of tRNA-Ser (GCT) gene in 4 Peristediidae mitogenomes. Note: (a) *P. liorhynchus*, (b) *S. amiscus*, (c) *S. rieffeli*, (d) *S. welchi*.
